# Mangrove Forests: Natural Laboratories for Studying Epigenetic and Climate Changes

**DOI:** 10.3389/fpls.2022.851518

**Published:** 2022-02-22

**Authors:** Matin Miryeganeh

**Affiliations:** Plant Epigenetics Unit, Okinawa Institute of Science and Technology Graduate University, Okinawa, Japan

**Keywords:** mangroves, plasticity, DNA methylation, transposable elements, adaptation

## Abstract

In the adaptation market, plants cash in the changes of their DNA (either genetic or epigenetic) to purchase fitness. Fitness is negatively affected by stressful conditions caused by climate change and well-designed studies are required to investigate the fine-tuning cooperation of epigenetic and genetic changes in response to those stresses. Mangrove trees are promising model systems for studying climate change because the effects of environmental changes are already evident in their natural habitats where they are exposed to different salinity levels ranging from saltwater to freshwater. In addition, as mangrove species are known to have very low genetic diversity caused by their stressful living conditions, epigenetic variation is likely to be a vital source for them to respond to environmental changes. This mini review aims to provide an overview of available studies on epigenetic regulation and adaptation of mangroves.

## Introduction

Mangrove trees play an important role in serving the ecosystem. They protect lands against erosion, provide nursery for sea creatures, filter pollutants from water, and store more carbon in the soil beneath them than even the rainforests. Unfortunately, it is predicted that within the next 80 years, the world is likely to lose the support of mangroves (Duke et al., 2007). Besides human intervention, climate change is one of the main threats that is putting one in five mangrove species in danger of extinction according to IUCN Red List of Threatened Species ™. Generally, the impact of climate change on plant species depends on the degree of their ability to acclimate and adapt to those changes. For example, individuals of a population that express genes related to stress-tolerance, can cope better with environmental extremes, surviving longer, and having more offspring. This means that over time, specific stress-tolerance traits encoded in the DNA may be preserved through natural selection. However, selection requires time, and the ongoing rapid pace of climate change makes it difficult for plants to keep up with the change and adapt fast enough before they undergo irreversible damages. This is where epigenetic regulation, which refers to the structural and chemical modifications on the genome without affecting the underlying DNA sequences, comes to rescue them. Epigenetic adjusts the expression of the genes in response to environmental signals and is known to be the main tool for acclimation in plants (Chano et al., 2021). Plants cannot change migration patterns or behavior like animals to adapt to environmental changes. Therefore, epigenetic responses and the pattern of expression of stress response genes could be very critical for their survival.

**Figure 1:**
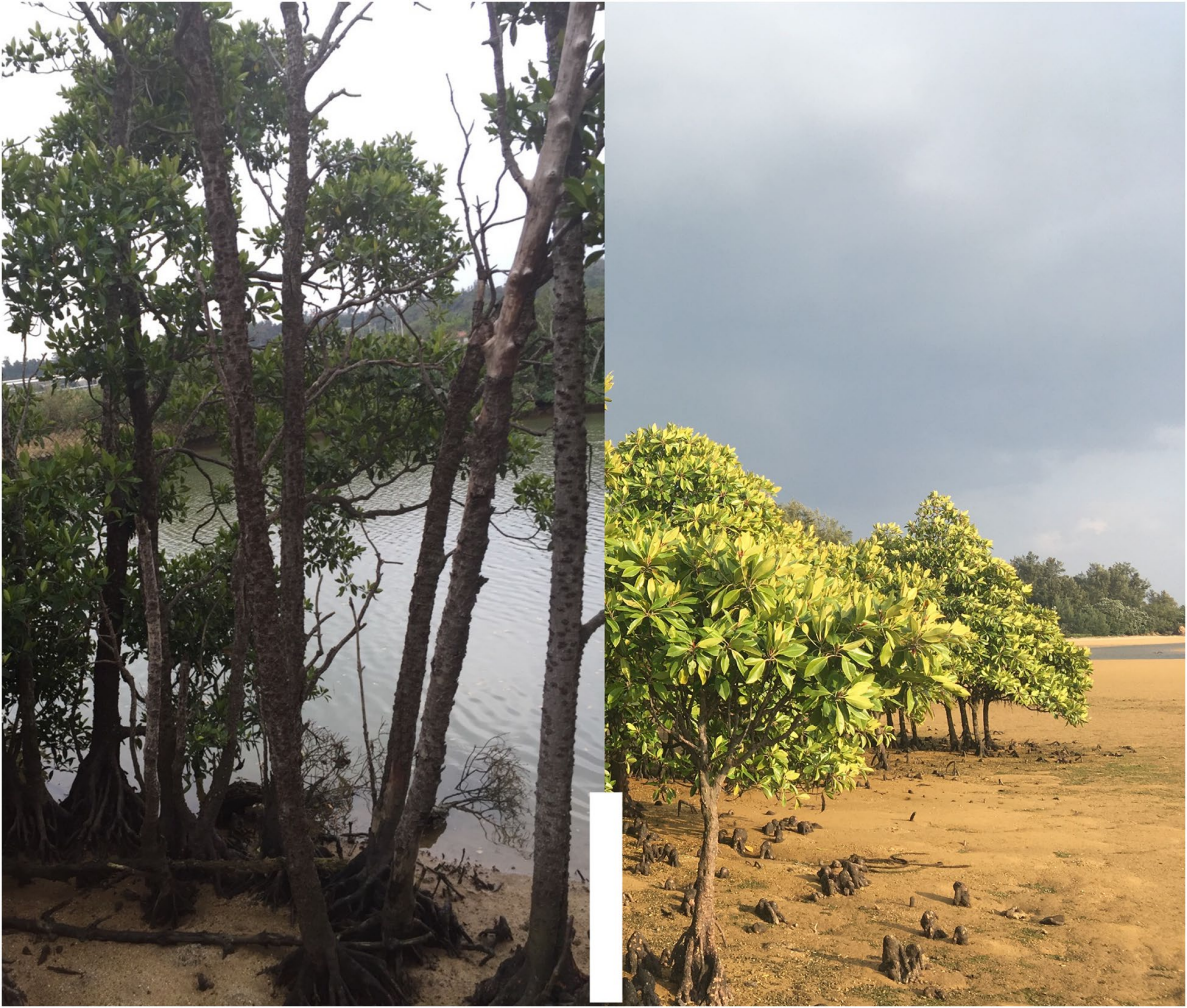
Two populations of a mangrove tree (*Bruguiera gymnorhiza*) growing in their natural habitat only about 1 km apart from each other. One population (left) located along the estuary of the Okukubi River (Kin town, Okinawa Island, Japan: 26°27′N, 127°56′E), in fresh/brackish water, showing tall tree-like morphology, and the other population (right) grow, in the coastal area of Pacific Ocean in saline environment, showing shrub-like phenotype. The scale bar marks about 100 cm. This is a typical characteristic of mangrove community. They usually show gradient morphological changes according to their environment ranging from fresh to saline water which makes them a natural laboratory for studying climate change and epigenetic mechanisms.

Epigenetic changes are either “stable” or “inducible.” Stable epigenetic modifications are those that serve the adaptation purposes in a similar way as the DNA sequence itself while inducible epigenetic markers are those that change during the lifetime of an individual and have the potential to be transgenerational, meaning the trait could be passed on to the next generation (also known as stress memory; Miryeganeh and Saze, 2020; Miryeganeh, 2021). This is quicker than nucleotide mutation and therefore may facilitate survival at the time of rapid environmental changes. It is important to investigate the mechanisms by which these stable and inducible markers contribute to adaptation. Whether and how epigenetic modification of the genome allow plants to respond to climate change in a more efficient way than natural selection and whether the resulted phenotypic plasticity from epigenetic changes is transgenerational and can be—at least partially—transferred to the next generation are ongoing investigations. Mangrove trees make an ideal (experimental) model system for studying the role of epigenetic mechanisms in adaptation to the chronic and fluctuating stress caused by climate change, because in their natural habitats they are already dealing with daily fluctuation of stresses such as salinity caused by tidal changes. Mangroves populations usually show gradient morphological changes relative to their distance from the sea, ranging from saline to brackish/fresh water (Figure 1). Furthermore, it is known that mangrove species have a reduced genetic diversity background which is suggested to be related to their stressful environment (Duke, 2011), and therefore epigenetic variation is a vital source for them to respond to environmental stress. In addition, as saline soil with little or no oxygen, is not a conducive environment for seeds to germinate and establish, many mangrove species have developed a unique way of reproduction, called vivipary, where seeds germinate and develop into seedlings (propagules) while are still attached to the parent tree that provide necessary supplies such as water and nutrients for them. This unique vivipary trait of mangroves also provides the possibility of studying transgenerational epigenetic diversity by sampling propagules that are still attached to the maternal plant.

Unfortunately, research on the connection between epigenetic regulation and adaptation of mangroves is very rare, mainly because of the lack of genome references for most of these species. One of the first epigenetic studies on phenotypic plasticity of mangroves was conducted when (Lira-Medeiros et al., 2010) investigated the potential effect of DNA methylation on two nearby populations of a mangrove species (*Laguncularia racemosa*) in response to high salinity stress. Despite of great morphological differences, individuals of natural populations from habitats with different salinity levels showed low genetic- but high epigenetic-differentiation, suggesting that DNA-methylation variation among individuals with similar genetic profile may cause phenotypic variation in response to different environments. However, this study was based on sampling cytosine methylation using methylation-sensitive amplification polymorphism (MSAP) technology, a PCR-based restriction-fragment length polymorphism method that monitors differential cleavage of methylation-sensitive restriction enzymes. In a more recent study (Mounger et al., 2021) investigated the pattern of DNA nucleotide sequence (genetic diversity) and DNA methylation (epigenetic diversity) in natural populations of a mangrove species *Rhizophora mangle*. They reported low genetic diversity, yet high epigenetic diversity among natural populations. Moreover, in a common garden experiment using offspring of the maternal trees from the natural population, they found that about 25% of epigenetic differences among offspring could be explained by maternal plants. The footprint of DNA methylation in the offspring plants implies that, to some extent, epigenetic variation was inherited from the maternal plant/environment even after growing under a new environmental condition. Their study provided some of the first evidence for epigenetic memory in mangroves. However, they also measured genetic and DNA methylation differentiation among populations using the small proportion of the genome sampled with the reduced representation bisulfite sequencing (RRBS) approach epigenotyping by sequencing (Van Gurp et al., 2016). Although both of these techniques (MSAP and RRBS) have the advantage of sampling sequences across a genome without prior sequence knowledge, studying cytosine methylation at single nucleotide resolution based on high-throughput sequencing of, e.g., bisulfite-treated genomic DNA which requires a high-quality reference genome sequence will generate richer datasets that along with transcriptome analysis, will bring insights to the molecular basis and the epigenetic story behind adaptation of these plant species (Richards, 2011). As mentioned above the lack of genome references for most of the mangrove species has made this path more challenging and time-consuming. Fortunately, there have recently been promising progresses in this area that are discussed in this mini review.

## DNA Methylation and Stress-Response in Mangroves

Environmental signals are known to change DNA methylation level, which is usually associated with the variation of gene expression, and together they could facilitate adaptation. Studying three main mangrove taxa (i.e., *Avicennia marina*, *Rhizophora apiculata*, and *Sonneratia alba*), Wang et al. (2021a) suggested that gene body methylation in mangroves are evolutionarily maintained by natural selection and has caused transcriptional robustness (i.e., low variation in gene expression) independent of genetic variation. They reported *de novo* gene body methylation in these mangrove species, which was convergently acquired after diverging from their common ancestors. The comparison between the role of genetic and epigenetic variation in their response to long-term stress showed minimum overlap between genes that were convergently methylated, and those with convergent DNA-sequence variation, which suggested an independent role for epigenetic in adaptation of mangroves to stress (Wang et al., 2021a). They further found decrease of changes in gene expression associated with DNA methylation (either stress-induced or evolutionarily gained). Their study led to the conclusion that transgenerational inheritance of the obtained gene body methylation facilitates the adaptation of mangroves to chronic stress through which they also experienced a major decrease in genetic diversity. The possible transgenerational inheritance of such methylation can have an important effect on adaptation of mangroves that generally are known to have low genetic diversity. This could explain the previously reported transcriptional homeostasis and robust transcriptional regulation (i.e., stable status in global transcriptional changes) of mangrove species under salt stress (Liang et al., 2012). In addition, it has been reported that mangroves that are halophyte species, living in high salinity, have fewer stress-responsive genes compared with glycophytes that live in mild salinity (Dassanayake et al., 2009) which implies a role for selection in their transcriptional robustness.

The genome of mangroves are the smallest among tree species (Xu et al., 2017; Hu et al., 2020), which is probably due to convergent reduction of transposable elements (TEs, that are repetitive regions of the genome) load (Lyu et al., 2018a,b). This may explain the reported expression of RNA directed DNA methylation pathway genes that suppresses the accumulation of TEs in the genome (Wang et al., 2018). In a recent study Miryeganeh et al. (2021) reported genome-wide DNA hypermethylation of TEs in mangroves growing under high salinity conditions which was associated with transcriptional regulation of chromatin modifier genes, suggesting robust epigenome regulation of TEs in the genome. Using their newly assembled genome of one of the most widely distributed mangrove species *Bruguiera gymnorhiza* they studied epigenetic signatures of high salinity-stress using two populations of this species in Okinawa, Japan that showed outstanding phenotypic differences depending on their environmental conditions. Trees that lived next to the ocean in saline condition displayed a dwarfed, phenotype with smaller leaves, while those growing in brackish condition along a river—only about 1 km away—were 10 times taller with larger leaves and thicker trunks (Figure 1). To understand the molecular mechanisms behind these morphological differences, they investigated the association between genome-wide differential gene expression (DGE) and DNA methylation profiles of the two saline and brackish populations. Thousands of differentially expressed genes were found, including genes known to be involved in saline response and the results were verified in a control laboratory experiment where seedlings were grown under saline and brackish condition mimicking the natural environments. They also found thousands of differentially methylated regions (DMRs) throughout the genome which was found mainly as cytosine hypermethylation of CHH and CHG sites (where H = A, T or C) in TEs among saline individuals in both natural and controlled experiments (Miryeganeh et al., 2021).

Despite the small genome and low TE proportion in mangroves, the studied mangrove species (*B. gymnorhiza*) was found to have the largest genome with the highest TE proportion among other reported species from the same family (Rhizophoraceae). This suggested that the loss of TEs in mangroves could be an evolutionary trait during their adaptation. *B. gymnorhiza* is the oldest in the family Rhizophoraceae, and was the first species to diverge from non-mangrove species in this family (Guo et al., 2017; Xu et al., 2017), and its phylogenetic position is thus isolated from other mangroves (Schwarzbach and Ricklefs 2000; Guo et al., 2017). Bigger genome and more TEs of this old species suggest that over time mangroves may have become stricter in silencing TEs by epigenetic regulation, in their attempt to adapt to environmental changes (Miryeganeh et al., 2021). Another interesting finding was the connection of genic DMRs to gene expression of several genes encoding BRUP-domain containing proteins (which has been named for the four members of the group initially identified, BNM2, USP, RD22, and PG1beta) present as a cluster of genes. These proteins are known to be downregulated in response to salt stress conditions (Miyama and Hanagata, 2007). Interestingly, there were many TEs, and other repeats located in this gene cluster which were also heavily hypermethylated in plants growing in saline conditions. This suggest that the methylated TEs may be involved in adaptation of mangroves in response to high salinity (Chuong et al., 2017; Johannes, 2021). Epigenetic control of TEs usually changes the expression of neighboring genes that may then cause phenotypic plasticity, which affects adaptive responses of mangroves to the harsh environment. It has been shown before that DNA methylation plays an important role in response of mangrove species to abiotic stresses such as high UV-B (Wang et al., 2021b). In addition, UV-B induced relaxation of TE silencing and therefore up-regulation of the TE-neighboring genes in mangrove species was reported which led to the conclusion of divergent clue behind UV-B adaptation at both the epigenetic and transcriptional level (Wang et al., 2021b). Recent transcriptional studies also have reported that in mangrove populations growing next to the ocean under high salinity various epigenetic regulation genes are differentially expressed along with upregulated stress response genes (Miryeganeh and Saze, 2021a, b). Studying epigenetic regulation in mangroves could be a strong equipment to find strategies for helping plants to cope with climate changes.

## Discussion

Mangrove forests could serve as natural laboratories for climate change research as the effects of environmental stress are already visible in their structural differences as they adapt to different salinity levels (Suwa et al., 2008; Miryeganeh et al., 2021). Mangrove communities usually show gradient morphological differences according to the level of salinity and other coastal stresses in their natural environments. Recent epigenetic studies have suggested that this gradient phenotypic changes is likely to be linked to the adaptation of mangroves rather than just their passive response to stress (Miryeganeh et al., 2021). Unfortunately, research on epigenetic regulation in mangroves is very rare, and hopefully drawing attention to this direction will stimulate more research in this area. Studies involving two consecutive generations, could reveal whether and to what extent epigenetic changes may improve the response of the next generation to environmental stress? Another question is if plants eventually will reach their limits to respond to climate change, even with epigenetic modes of adaptation; something that has been reported in fish (Heckwolf Melanie et al., 2020). It is also important to understand if transgenerational epigenetic modifications triggered by climate change necessarily increase fitness. The fact that epigenetic modifications come to rescue organisms from having a fixed destiny defined by their genes, brings hope to chance of survival of organisms, even if the original genetic sequences were not enough to save them. However, even though this sounds exciting and promising, the remaining question is if epigenetic changes are an adequate replacement for dealing with the current rapid-pace climate changes? Is it possible that epigenetic changes that were forced to be made in face of climate change cause more suffering and damage in the long run as it was just an act of quick fix for survival and not according to the well-planned natural selection of changing DNA sequences? Future studies using the natural laboratory of mangroves could help finding clues for these questions.

In addition, the reported BRUP cluster for the mangrove species *B. gymnorhiza* would make a promising starting point to study molecular mechanisms behind phenotypic plasticity studies of mangrove trees caused by high salinity. Searching syntenic regions of the BURP gene cluster between *B. gymnorhiza* and another close related mangrove species (*Kandelia obovata*) from the same family revealed that only a single BURP domain protein gene is encoded in the corresponding region of the other species (Miryeganeh et al., 2021). This suggest a rapid evolving of this cluster that may play an important role in adaptive evolution of mangroves and needs to be investigated further in future studies. The presence of many TEs within this cluster also suggests that repeat elements may have had an important role in forming BURP cluster as it has been reported for other gene families in other species before (Galindo-Gonzalez et al., 2017). Future studies focusing on the relationship between differential methylation patterns, and/or variations of repeat elements within BURP cluster and adaptational plasticity among populations of mangrove species could open a new avenue in exploring evolutionary history of mangroves and their adaptation to harsh environment. Identifying the association between differential methylation found within repeat elements and DGE changes at the genome-wide scale is a challenging task not only in mangroves but also in most plant species. One main reason could be that the DMR-DGE relationship may occur not only through cis, but also *via* distal regulatory interactions (Lu et al., 2018; Johannes 2021) for example through chromatin looping and distal regulatory elements (enhancers) as it has been reported in maize (Xu et al., 2020), or trans-acting small RNAs (Chuong et al., 2017). To detect these kinds of distal regulatory factors and their target genes, future studies that give us access to chromatin interaction maps and chromosome level assembly are critically needed. This requires isolating pure, intact, and high-molecular weight DNA from these species which is a challenging task for mangroves as they are specially adapted to extra harsh condition such as marshy anoxic anaerobic soil and fluctuating salinity in their surrounding soil and water. In response to this difficult environmental condition, mangrove synthesize a wide range of polysaccharides and polyphenols including flavonoids and other secondary metabolites such as alkaloids which interfere with the extraction of pure high molecular weight genomic DNA (Kathiresan and Bingham, 2001; Bandaranayake, 2002). Recent reports of long read sequencing of mangroves’ genome brings hope for future studies with even more improvement and accuracy that may give us access to chromosome level genome assembly and open a whole new avenue of possibilities for investigating the molecular regulatory system behind adaptation of these valuable tree species that can serve as experimental models for studying adaptation of plants in face of climate change.

## Author Contributions

The author confirms being the sole contributor of this work and has approved it for publication.

## Funding

Open access funding was provided by Okinawa Institute of Science and Technology (OIST) Graduate University.

## Conflict of Interest

The author declares that the research was conducted in the absence of any commercial or financial relationships that could be construed as a potential conflict of interest.

## Publisher’s Note

All claims expressed in this article are solely those of the authors and do not necessarily represent those of their affiliated organizations, or those of the publisher, the editors and the reviewers. Any product that may be evaluated in this article, or claim that may be made by its manufacturer, is not guaranteed or endorsed by the publisher.
